# Supplementary material for: Climate change-induced shifts in survival and size of the worlds’ northernmost oviparous snake: A 68-year study
Source: PLoS One. 2024 Mar 21;19(3):e0300363. doi: 10.1371/journal.pone.0300363 (PMC10956784; doi:10.1371/journal.pone.0300363)
Supplement: S1 Table — Details of the five best-fitting CJS models of recapture probability in a population of Grass snakes (Natrix natrix), ranked by AICc. (DOCX) [file pone.0300363.s001.docx]

**Supporting information**

Table S1. Details of the five best-fitting CJS models of recapture probability in a population of Grass snakes (*Natrix natrix*), ranked by AICc.

|  | Model | AICc | ΔAICc | $w_{i}$ | No. Par | Deviance |
| --- | --- | --- | --- | --- | --- | --- |
| 1 | p~ sex + effort + season + SVL | 3116.4 | 0.0 | 1.0 | 12 | 3092.1 |
| 2 | p~ sex + effort + SVL | 3148.9 | 32.5 | 0.0 | 11 | 3126.7 |
| 3 | p~ sex + season + SVL | 3156.6 | 40.2 | 0.0 | 11 | 3134.4 |
| 4 | p~ season + effort + SVL | 3159.9 | 43.6 | 0.0 | 11 | 3137.7 |
| 5 | p~ season + SVL | 3185.5 | 69.1 | 0.0 | 10 | 3165.3 |
