# Supplementary material for: Climate change-induced shifts in survival and size of the worlds’ northernmost oviparous snake: A 68-year study
Source: PLoS One. 2024 Mar 21;19(3):e0300363. doi: 10.1371/journal.pone.0300363 (PMC10956784; doi:10.1371/journal.pone.0300363)
Supplement: S2 Table — Details of the 10 best-fitting CJS models of survival over time, sex, and season in a population of Grass snakes (Natrix natrix), ranked by AICc. (DOCX) [file pone.0300363.s002.docx]

**Supporting information**

Table S2. Details of the 10 best-fitting CJS models of survival over time, sex, and season in a population of Grass snakes (*Natrix natrix*), ranked by AICc.

| Model | | | AICc | ΔAICc | $w_{i}$ | No. Par | Deviance |
| --- | --- | --- | --- | --- | --- | --- | --- |
| 1 | φ~ sex + SVL + season + year + year:season | | 3065.7 | 0.0 | 0.557 | 11 | 3043.4 |
| 2 | φ~ sex + SVL + season + year + sex:season + year:season | | 3066.3 | 0.6 | 0.407 | 12 | 3042.0 |
| 3 | φ~ sex + SVL + season + sex:season | | 3073.4 | 7.7 | 0.012 | 10 | 3053.2 |
| 4 | φ~ sex + SVL + season | | 3073.4 | 7.7 | 0.012 | 9 | 3055.2 |
| 5 | φ~ sex + SVL + year | | 3073.9 | 8.2 | 0.009 | 9 | 3055.7 |
| 6 | φ~ sex + SVL | | 3076.0 | 10.4 | 0.003 | 8 | 3059.9 |
| 7 | φ~ SVL + season + year + year:season | | 3101.0 | 35.4 | 0.000 | 10 | 3080.8 |
| 8 | φ~ SVL + season + year | | 3108.4 | 42.8 | 0.000 | 9 | 3090.3 |
| 9 | φ~ SVL + season | | 3109.9 | 44.3 | 0.000 | 8 | 3093.8 |
| 10 | | φ~ SVL + year | 3113.2 | 47.5 | 0.000 | 8 | 3097.1 |
