# Supplementary material for: Climate change-induced shifts in survival and size of the worlds’ northernmost oviparous snake: A 68-year study
Source: PLoS One. 2024 Mar 21;19(3):e0300363. doi: 10.1371/journal.pone.0300363 (PMC10956784; doi:10.1371/journal.pone.0300363)
Supplement: S3 Table — Details of the 10 best-fitting CJS models of survival with climatic data in a population of Grass snakes (Natrix natrix), ranked by AICc. (DOCX) [file pone.0300363.s003.docx]

**Supporting information**

Table S3. Details of the 10 best-fitting CJS models of survival with climatic data in a population of Grass snakes (*Natrix natrix*), ranked by AICc.

| Model | | | AICc | ΔAICc | $w_{i}$ | No. Par | Deviance |
| --- | --- | --- | --- | --- | --- | --- | --- |
| 1 | φ~ sex + SVL + rain w | | 3072.6 | 0.0 | 0.167 | 9 | 3054,4 |
| 2 | φ~ sex + SVL + rain s + snow depth | | 3073.2 | 0.7 | 0.120 | 10 | 3053.0 |
| 3 | φ~ sex + SVL + snow depth | | 3073.9 | 1.3 | 0.085 | 9 | 3055.7 |
| 4 | φ~ sex + SVL + rain w + rain s | | 3074.5 | 1.9 | 0.063 | 10 | 3054.3 |
| 5 | φ~ sex + SVL + rain s + snow depth + snow interval | | 3075.1 | 2.5 | 0.048 | 11 | 3052.8 |
| 6 | φ~ sex + SVL + temp s + snow depth | | 3075.1 | 2.5 | 0.047 | 10 | 3054.9 |
| 7 | φ~ sex + SVL + temp w + rain s | | 3075.2 | 2.6 | 0.046 | 10 | 3055.0 |
| 8 | φ~ sex + SVL + rain s | | 3075.4 | 2.9 | 0.041 | 9 | 3057.2 |
| 9 | φ ~ sex + SVL + temp w + rain w + rain s | | 3075.4 | 2.8 | 0.040 | 11 | 3053.2 |
| 10 | | φ~ sex + SVL + temp s + rain w | 3075.6 | 3.0 | 0.037 | 10 | 3055.4 |
